# Continuities and change in alcohol policy at the global level: a documentary analysis of the 2010 Global Strategy for Reducing the Harmful Use of Alcohol and the Global Alcohol Action Plan 2022–2030

**DOI:** 10.1186/s12992-024-01034-y

**Published:** 2024-06-14

**Authors:** Matthew Lesch, Jim McCambridge

**Affiliations:** 1 Department of Politics and International Relations, Derwent College, University of York, D/N/126, Heslington, York, YO10 5DD UK; 2 Department of Health Sciences, University of York, York, UK

**Keywords:** Alcohol policy, Global health, Global governance, Alcohol industry, Commercial determinants of health, Public health

## Abstract

**Background:**

There are only two major statements which define alcohol policy development at the global level. There has not been any comparative analysis of the details of these key texts, published in 2010 and 2022 respectively, including how far they constitute similar or evolving approaches to alcohol harm.

**Methods:**

Preparatory data collection involved examination of documents associated with the final policy statements. A thematic analysis across the two policy documents was performed to generate understanding of continuity and change based on comparative study. Study findings are interpreted in the contexts of the evolving conceptual and empirical literatures.

**Results:**

Both documents exhibit shared guiding principles and identify similar governance challenges, albeit with varying priority levels. There is more emphasis on the high-impact interventions on price, availability and marketing in 2022, and more stringent targets have been set for 2030 in declaring alcohol as a public health priority therein, reflecting the action-oriented nature of the Plan. The identified roles of policy actors have largely remained unchanged, albeit with greater specificity in the more recent statement, appropriately so because it is concerned with implementation. The major exception, and the key difference in the documents, regards the alcohol industry, which is perceived primarily as a threat to public health in 2022 due to commercial activities harmful to health and because policy interference has slowed progress.

**Conclusions:**

The adoption of the Global Alcohol Action Plan 2022-30 potentially marks a pivotal moment in global alcohol policy development, though it is unclear how fully it may be implemented. Perhaps, the key advances lie in advancing the ambitions of alcohol policy and clearly identifying that the alcohol industry should not be seen as any kind of partner in public health policymaking, which will permit progress to the extent that this influences what actually happens in alcohol policy at the national level.

## Introduction

Alcohol has garnered increased attention at the global health level in recent decades [1, 2]. There is a growing consensus in public health that global cooperation led by the World Health Organization (WHO) is needed to support the national policy changes that are required to address alcohol effectively as a public health issue [3–5]. The WHO has had a long-standing interest in reducing alcohol harms, playing key roles in developing scientific principles and evidence since the 1950s [3]. More recently, however, it has actively sought to establish a more explicit policy role, drawing the global community’s attention to the health, social, and economic burden of alcohol and how it can be reduced. These efforts have been vital in securing the inclusion of alcohol on the agendas of the World Health Assembly (WHA), the WHO’s key decision-making body.

Internationally, individual states and groups of Member States have proven to be influential players in advancing these global efforts [6]. Recent studies have shown that global institutions, particularly the WHO, are key sites of political contestation between the industry and public health actors over alcohol policy [7–13]. There is, however, a paucity of studies of how approaches to global alcohol policy development have actually shifted in substance in recent years [14].

Alcohol was first discussed during the fifty-eighth WHA in 2005. The Director-General was requested to report to the sixtieth WHA on “evidence-based strategies and interventions to reduce alcohol-related harm, including a comprehensive assessment of public health problems caused by harmful use of alcohol” [15]. This led to the convening of the WHO Expert Committee on Problems Related to Alcohol Consumption to review the evidence on alcohol-related harms and potential policy responses [16]. The WHA did not reach a consensus on a strategy in 2007 and instead recommended consultation to facilitate the development of a strategy by 2010 [17]. Following several rounds of consultation, the WHO Executive Board (EB) finalised the global strategy in January 2010. The Global Strategy for Reducing the Harmful Use of Alcohol (hereafter Global Global Strategy (GS)) was adopted at the WHA?s sixty-third session in May 2010 [2]. The GS provided guidance to Member States on ways to reduce the harmful use of alcohol [18]. Several WHO regional offices have since developed or revised regional strategies to align with the GS [for example: 19, 20].

Policy development by Member States has been decidedly slow and uneven. According to the WHO assessment in 2018, limited progress had been made in tackling alcohol-related harm, including on the key indicator of reduced per capita consumption [21]. Some attribute the strategy’s limited progress to its recommendations lacking “clear targets and specific goals” [22]. Moreover, implementation has been slowest for the most effective measures which reduce availability, affordability and marketing, and in low- and middle-income countries where it may be particularly needed.

Several efforts have been underway to overcome governance obstacles to implementation. First, the SAFER initiative was launched in 2019, aimed at promoting the adoption of the most cost-effective and under-used interventions. SAFER was designed to promote the implementation of high-impact policies – most importantly, pricing, retail and marketing interventions [23]. Second, as SAFER was not regarded as sufficient by itself to achieve the 2010 target of a 10% reduction by 2025, further actions were deemed to be needed, and key developments subsequently unfolded at the political level.

In February 2020, in its resolution, EB146 [18], the EB advocated for accelerated action to reduce the harmful use of alcohol. It called on the WHO Director-General “to develop an action plan (2022–2030) to effectively implement the Global strategy to reduce the harmful use of alcohol as a public health priority, in consultation with Member States and relevant stakeholders” [24]. The decision to develop an action plan also stemmed from an unsuccessful proposal to the EB by some low- and middle-income countries seeking to establish a working group tasked with reviewing the feasibility of an international treaty on alcohol control, akin to the Framework Convention on Tobacco Control (FCTC) [25]. The draft action plan was approved by the EB and adopted by the seventy-fifth WHA in 2022. The Global Alcohol Action Plan (GAAP) includes more ambitious targets for reducing alcohol consumption and specifies action areas in which to concentrate implementation efforts. The revised targets include a 20% reduction from a 2010 baseline in per capita consumption by 2030, in alignment with the Sustainable Development Goals architecture.

These policy developments have occurred alongside key advances in the evidence base on alcohol policy. First, there is broad scientific consensus that population-level approaches, including controls on alcohol pricing [26, 27], advertising [28–30], and availability [31] are needed to reduce alcohol-related harm. Recent analyses of policy developments in Scotland [32], Ireland [33], Lithuania [34] and Estonia [35] demonstrate the importance of this evidence base in facilitating policy change. Second, researchers have identified the alcohol industry as a key barrier to the enactment and implementation of evidence-informed policies at the national level [36, 37–40]. The evidence-base has expanded rapidly in recent years, with studies adding greater depth to our understanding of how industry actors have effectively mobilised to oppose alcohol policy development and implementation across the world [7, 9, 10, 41, 42]. There have also been significant advances in some countries, providing important lessons on how industry opposition may be overcome [32, 33, 42, 43–51].

Several conceptual frameworks have been developed to better understand the nature, scope, and complexity of industry power in policymaking [52–54]. Another emerging framework, the commercial determinants of health (CDoH), specifies the system-level factors, underlying ideas, and commercial practices that perpetuate commercial influence [55]. The latter encompasses several different political (e.g., lobbying), scientific (e.g., funding research), and reputational practices (e.g., corporate social responsibility) that promote commercial interests [55]. The nature of these practices is variable between commercial sectors and companies within sectors, with all such practices having capacity to steer policy decisions at global, regional and national levels, particularly for the largest companies and sectors [56–61]. This literature is particularly relevant for well consolidated health-harming commercial sectors such as alcohol [55].

This study explores the development of global alcohol policy against the backdrop of recent developments permitting more nuanced understanding of the alcohol industry and its political activities. This study provides the first systematic comparison of the GS and GAAP from a public health perspective. These two policy documents serve as the fundamental framework for alcohol policy globally. Therefore, our analysis aims to identify whether and how global approaches to alcohol policy have evolved, while also highlighting significant consistencies, over time. This investigation thus examines comparatively the substantive content of the two texts, with interpretation informed by the evolving conceptual and empirical literatures.

## Methods

Data analysis proceeded in four main steps. First, we familiarised ourselves with the contexts surrounding the adoption of the GS and GAAP by collecting and analysing key precursor documents and associated literature. In so doing, we consulted the limited research on alcohol policy development at the global level [e.g., 8–10, 22, 62–69].

Second, the first author generated summaries of the two policy documents that constitute the core object of study. This involved a high-level description of the key aims and scope of each document. This material was discussed to better contextualise and orientate the interpretation of each document, and later their comparison. The descriptive content, as well as the discussion notes, were summarised in one document. Our report of the findings includes several direct quotations which are drawn from the original two policy documents. In addition, we identify the proposed action areas for Member States and highlight selected examples of these proposed actions for illustrative purposes. These examples were included based on their relevance to the content we analysed and presented (Results) and to our broader discussion of the findings (Discussion).

Third, the first author undertook a more detailed comparison of the two documents by examining how each described and presented alcohol as a policy challenge, the core policy aims, the types of language used, and the specified actions. The dataset was created by extracting the key text from each document. These data were then placed into NVivo to facilitate thematic analysis. This comparative technique follows a similar approach employed by O’Brien and colleagues in their analysis of the alcohol industry’s submissions to consultations on the GAAP [8]. To examine the influence of industry arguments on policy, these authors compared the initial draft of the GAAP and the final version of the GAAP, examining the possible effects of industry arguments as found in the first publicly available consultation dataset [8].

Finally, we used thematic analysis to refine our interpretation of the content, seeking to identify key themes across the documents relevant to comparative study [70]. Codes were generated inductively through analysis of the two documents. The first author led all stages of the analytic process with the second author playing a supporting role.

**Fig. 1:**
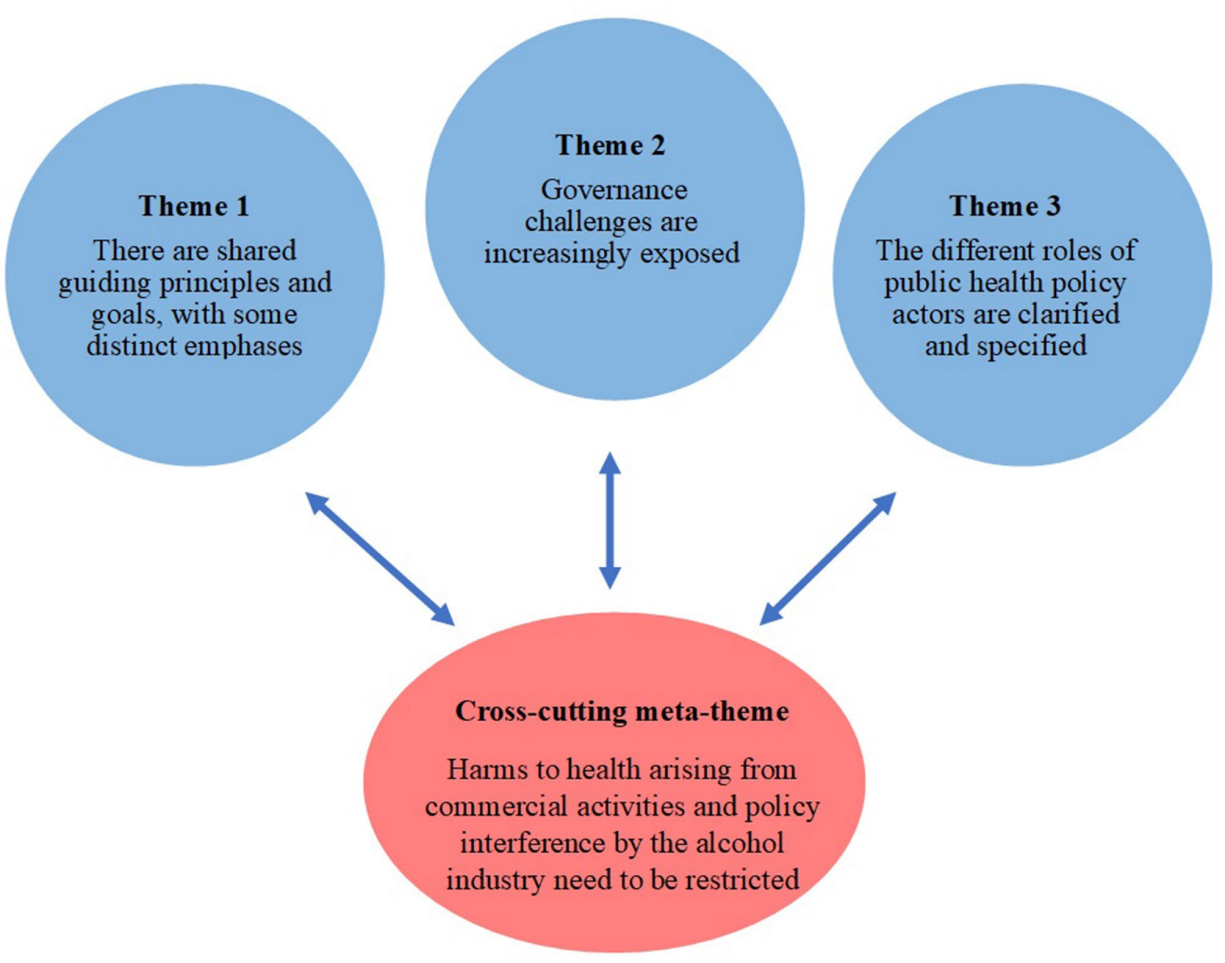
Overview of core themes and cross-cutting meta-theme

This process resulted in the identification of a set of core themes and a cross-cutting meta-theme (see Fig. 1). We also identified several subthemes and assessed the strength of the connection (strong, moderate, or weak) between each sub-theme and the cross-cutting meta-theme (see Table 1). The assessed strength of the connection to the meta-theme reflects the extent to which the sub-theme is impacted by the distinct approach taken to industry actors in the GAAP. The final analysis, which subsequently involved the refinement of the thematic content, is provided in this report.

**Table 1:** Summary of thematic findings

Core theme	Subtheme	Connection of sub-theme to cross-cutting meta-theme across the GS and GAAP
Theme 1	Public health interests should guide the design of policies and interventions	Strong
Theme 1	Alcohol policies should be flexible and sensitive to different contexts (e.g., equity, national and cultural considerations)	Moderate
Theme 1	The responsibility of different policy actors to work together for public health	Moderate
Theme 1	The need for principle-centred approaches to alcohol policymaking	Moderate
Theme 1	Equity as a key principle and governing challenge	Weak
Theme 2	The importance of global action and international cooperation	Strong
Theme 2	The need to increase decision-makers’ attention to alcohol-related harm	Strong
Theme 2	The need for intersectoral policy action	Strong
Theme 2	Promotion of partnerships	Moderate
Theme 2	Weak information systems regarding alcohol-related harm as a systematic barrier	Weak
Theme 2	The need for strengthening policy capacity via resources and stronger technical support	Weak
Theme 3	Member States as the key locus of decision-making on alcohol policy	Strong
Theme 3	WHO Secretariat as the coordinator, facilitator, and key technocratic actor in the policy process	Strong
Theme 3	Civil society and other entities as key actors in strengthening public awareness of alcohol-related harm	Strong

## Results

### The global strategy

Rather than determining policy, the GS establishes “priority areas for global action and “to recommend a portfolio of policy options and measures that could be considered for implementation” p.8”. As described below, there is considerable focus on providing flexibility for Member States in designing alcohol policies to align with their national contexts. Thus, the strategy provides a menu of options for areas of action aimed at reducing the harmful use of alcohol as follows:

- leadership, awareness and commitment.
- health services’ response.
- community action.
- drink-driving policies and countermeasures.
- availability of alcohol.
- marketing of alcoholic beverages.
- pricing policies.
- reducing the negative consequences of drinking and alcohol intoxication.
- reducing the public health impact of illicit alcohol and informally produced alcohol.

j) monitoring and surveillance.

The GS requires that each Member State conducts an internal assessment and devises a plan, along with a monitoring system, to effectively reduce alcohol-related impacts. Moreover, it urges countries to establish a permanent coordinating body, comprising senior representatives from relevant government departments, civil society, and professional associations. Furthermore, Member States are expected to develop clear and objective strategies tailored to local circumstances, incorporating measurable targets based on available evidence.

### The global alcohol action plan

The chief goal of the plan is to” boost the effective implementation of the global strategy to reduce the harmful use of alcohol as a public health priority and to significantly reduce morbidity and mortality due to alcohol consumption” (p.9).

The GAAP strives for 70% of countries to have “introduced, enacted or maintained the implementation [of]” several high-impact policy measures (p.12). These are the pricing, availability and marketing measures that are included within SAFER (along with drink driving and health sector responses) and the GS.

The GAAP identifies 6 key action areas, specifically:

- Implementation of high-impact strategies and interventions.
- Advocacy, awareness and commitment.
- Partnership, dialogue and coordination.
- Technical support and capacity-building.
- Knowledge production and information systems.
- Resource mobilization.

The GAAP places significant emphasis on the first action area, explaining how, if implemented, these high-impact strategies have “the highest potential” for reducing alcohol-related harm (p.8). Within the six action areas, the GAAP identifies specific leadership and/or supporting roles and specific actions for Member States, the WHO Secretariat, international partners, civil society organisations, and academics. Separately, there are a series of proposed measures for economic operators in alcohol production and trade in each of the 6 areas.

Figure 1 provides an overview of the main study findings. Table 1 presents the 14 sub-themes that emerged from the comparison of the two documents, providing a summary of the analytic material. It also identifies the strength of the connection between sub-themes and the meta-theme (see below).

## There are shared guiding principles and goals, with some distinct emphases

The main goal of the GS is to “promote and support local, regional and global actions to prevent and reduce the harmful use of alcohol” (p.8). A key focus of the GS is flexibility, explaining how policy options could be implemented and “adjusted as appropriate at the national level” (p.3). The GAAP shares many of the underlying goals while placing a greater emphasis on securing the implementation of the strategy’s goals. The goals of GAAP are thus more action-oriented. Moreover, the GAAP, unlike the GS, declares alcohol to be a public health priority and sets more ambitious targets (see above).

Both documents outline several core principles that exhibit both continuity and change. There is a broad consensus across the documents that public health interests should guide the design of policies and interventions (See Table 1). The GS asserts that policies and interventions should be “guided and formulated by public health interests” (p.9). GAAP takes a step further, emphasising the vital importance of “protecting public health-oriented policymaking against interference from commercial interests” (p.4). While the GS acknowledges the need to give “proper deference” to public health in relation to competing interests (p.9), this discussion is absent in the GAAP. Instead, the GAAP states that policy development “should be protected, in accordance with national laws, from commercial and other vested interests that can interfere with and undermine public health objectives” (p.11).

Sensitivity to context, including cultural and equity concerns, is also regarded as a key underlying principle shared by both documents. The GS maintains that alcohol policies need to be “equitable and sensitive to national, religious, and cultural contexts” (p.9). However, GAAP approaches this issue somewhat differently; “religious beliefs and spiritual and cultural norms… and commercial interests” have shaped the approaches taken by national and subnational governments in addressing alcohol-related problems (p.5). GAAP’s treatment of inequities also varies. It discusses prioritising the reduction of “health inequalities” and the need to “protect people in different groups […] from alcohol-related harm” (p.11).

The GS acknowledges the importance of contextual factors in potentially influencing the effectiveness of various alcohol policies. It emphasised the need for “appropriate tailoring” of policy interventions to ensure that “local contexts” are taken into account (p.7). On the other hand, GAAP places somewhat less emphasis on this aspect but still recognises the governance challenges posed by “differences in cultural norms and contexts” (p.5). In comparison to the GS, GAAP devotes less attention to issues related to local contexts. On the other hand, as noted above, there is considerably more attention paid to high-impact measures and the need to accelerate their implementation globally, as reflected in a specific target that 70% of all countries should be enacting such measures by 2030.

Another shared principle relates to the responsibility of different policy actors to work together. The GS states that “parties have the responsibility to act in ways that do not undermine the implementation of public policies and interventions to prevent and reduce harmful use of alcohol” (p.13). The GAAP reaffirms its commitment to this principle, asserting that national policies “should be guided and formulated by public health interests and based on clear public health goals and the best available evidence” (p.4). The GAAP goes beyond mere affirmation by detailing specific “enabling or recommended actions” for Member States (p.4). These actions encompass raising alcohol taxes, implementing and enforcing total bans or restrictions on different types of alcohol marketing, as well as limitations on the physical availability of retailed alcohol.

The GAAP identifies the need for principle-centred approaches to alcohol policy, such as life-course- and human-rights-based approaches. The epidemiologically-based former principle strives to ensure that interventions are designed “to prevent and reduce alcohol-related harm at all stages of a person’s life and for all generations” (p.10–11). This principle encompasses “the protection of the unborn child from prenatal alcohol exposure” to addressing “harms due to the use of alcohol in older people” (p.10). Regarding human rights, the emphasis is arguably somewhat restricted, on ensuring people have “access to the prevention and treatment of AUDs” and taking steps to eliminate “discriminatory practices (both real and perceived) and stigma” among this group (p.11). This nonetheless represents a change from the GS as there are no mentions of a life-course approach or human rights in the GS.

Following on from the above, equity is stated more prominently as a principle in the GS. The 2010 policy document discusses the need for policies and programs that could “reduce…social disparities both inside a country and between countries” (p.7). GAAP appears more concerned about what to do about equity issues, reflecting the action-oriented nature of the document. For example, it identifies “the need for more resources and greater priority to be allocated to support the development and implementation of effective policies and actions in low- and middle-income countries” (p.5). This indicates a focus on the overall allocation of resources for effective implementation. This suggests a growing recognition that beyond the initial principles and goals outlined in the GS, the level of resources invested by Member States needs to be increased.

## Governance challenges are increasingly exposed

In part due to the failure to implement the GS’s policy recommendations, problems of governance are a particularly explicit theme in the GAAP. Several characteristics inherent to alcohol policymaking are considered especially problematic and are substantiated by existing evidence. First, alcohol-related issues are often considered particularly complex and cross-cutting. It has long been acknowledged, for example, that at the domestic level, alcohol policymaking is marked by inter-departmental conflicts of interest [71]. Second, notable differences in cultural norms and contexts surrounding alcohol use, raise concerns about policy portability [72]. Third, the influence of powerful commercial interests presents a perennial barrier to evidence-informed alcohol policy change [73]. Finally, persistent challenges in obtaining comprehensive and reliable data on alcohol, particularly when compared to other health-harming products like tobacco [74], further underscore the complexities in addressing this issue.

Both the GS and GAAP stress the need to increase global action and international cooperation. This is identified as a particularly important challenge. In the case of the GS, “global guidance” and “increased international collaboration” are deemed imperative as they would help ensure that regional and national actions can work in unison (p.6). While focusing on a different dimension of coordination, the GAAP also prioritises this issue. It observes that policy implementation has been “uneven” across different regions and countries since the GS was ratified (p.5). Unlike the GS, however, the GAAP’s focus shifts toward identifying and tackling potential barriers to implementation. For example, it emphasises the need for “strong international leadership… to counter interference from commercial interests in alcohol policy development and implementation” (p.5). According to the GAAP, confronting the source of this problem is one of the most important ways to “prioritize the public health agenda for alcohol” (p.5).

Decision-makers’ limited attention to alcohol-related harm has persisted as a core governance concern. The GS highlights this as a major issue, noting how the burden of alcohol-related harm is given “low priority” despite “compelling evidence of its serious public health effects” (p.6). The GAAP’s continuation of this line of thinking suggests that political leadership continues to be a challenge. For example, the GAAP expresses concern that “awareness and acceptance of the overall negative impact of alcohol consumption on a population health and safety is low among decision-makers and the general public” (p.3) The GAAP itself, in declaring alcohol as a public health priority, alongside setting more ambitious targets, seeks to elevate attention to the governance issues.

The need for strengthening intersectoral policy action has persisted as an identified problem but has also been approached differently. The GS prioritises “appropriate engagement” with sectors beyond health, including non-health government departments, civil society groups, and “economic operators” (p.6). In contrast, the GAAP identifies “competing interests” within governments, as some departments may prefer lower alcohol taxes or less stringent regulations. The result often leads to “policy incoherence” (p.5). Public health leadership of alcohol policymaking to ensure health considerations are given due regard in all policies is increasingly important in this context.

The promotion of partnerships is a major governance priority in both documents. According to the GS, one of the main impediments to effective policy implementation is the lack of coordination within governments. It states that governments need to create “effective and permanent coordination machinery… to ensure a coherent approach to alcohol policies and a proper balance between policy goals in relation to harmful use of alcohol and other public policy goals” (p.10). In contrast, the GAAP highlights the commercial interests of alcohol producers (see below), whilst also giving attention to capacity issues.

The presence of weak information systems regarding alcohol-related issues continues to be a significant governance challenge in advancing policy implementation. Both documents highlight the existence of gaps in information systems, particularly concerning levels of alcohol consumption, associated harms, and data on policy measures, and command similar tone and language.

Technical support is also recognised as a related governance issue treated in similar ways in both documents. The GAAP devotes more attention to the need for resources and implies an urgency in the allocation of resources to enable actions to be taken within the timeframes set for the attainment of the key targets and indicators.

## The different roles of public health policy actors are clarified and specified

The more specific character of the content of the GAAP compared to the GS for the different types of public health policy actors is the key theme. This section necessarily presents a summary of the analytic comparisons made and indicative content only is provided here.

### Member states

There is broad agreement across the documents about the role of Member States. Specifically, both the GS and GAAP see Member States as the most important policymakers. The GAAP offers much more detailed expectations of Member States than laid out in the GS reflecting the nature of the document (i.e., an action plan). For example, Member States are expected to prioritise “the implementation and enforcement, continued enforcement, monitoring and evaluation of high-impact cost-effective policy options” (p.12). Table 2 presents example content on key Member State actions across the 6 areas that demonstrate specific features of the GAAP as distinct from the GS.

**Table 2:** The global alcohol action plan’s proposed actions for member states

Action area	Example of proposed action
1. Implementation of high-impact strategies and interventions	Action 1 – On the basis of the evidence of the effectiveness and cost-effectiveness of policy measures, to promote the prioritization, according to national needs and contexts, of the sustainable implementation, continued enforcement, monitoring and evaluation of high-impact cost-effective policy options included in the WHO SAFER technical package, as well as other interventions already proven to be cost-effective or subsequently proven to be cost-effective based on upcoming evidence, including the assurance of universal access to affordable treatment and care for people with AUDs within national health systems.
2. Advocacy, awareness and commitment	Action 8 – Ensure appropriate consumer protection measures through the development and implementation of labelling requirements for alcoholic beverages that display essential information for health protection on alcohol content in a way that is understood by consumers and also provides information on other ingredients with potential impact on the health of consumers, caloric value and health warnings.
3. Partnership, dialogue and coordination	Action 2 – Ensure effective national governance and effective coordination between different sectors and different levels of government, while maintaining policy coherence based on public health objectives.
4. Technical support and capacity-building	Action 2 – Develop national institutional capacities for applying population-wide initiatives to tackle the determinants that drive the acceptability, availability and affordability of hazardous and harmful drinking patterns, including for the provision of country-tailored technical assistance, strengthening governance mechanisms towards accountability, transparency and the participation of stakeholders.
5. Knowledge Production and Information Systems	Action 1 – Support the generation, compilation and dissemination of knowledge at the national level on the magnitude and nature of public health problems caused by the harmful use of alcohol and the effectiveness of different policy options, and undertake activities for informing the general public about health and other risks associated with alcohol consumption and alcohol-related health conditions in different populations.
6. Resource mobilization	Action 1 – Increase the allocation of resources, including international and domestic financial resources generated by new or innovative ways and means to secure essential funding, for reducing the harmful use of alcohol and increasing the coverage and quality of prevention and treatment interventions, according to the scope and nature of public health problems caused by alcohol consumption.

### WHO secretariat

The WHO Secretariat is expected to do technical work in both documents; to regularly prepare and disseminate global status reports on alcohol-related harm, develop and share technical and advocacy tools for effective policy communication, and facilitate knowledge exchange. GAAP is more specific about the responsibilities, and extends the role of the Secretariat, making the technical support more extensive. The GAAP, for example, expects the Secretariat to offer: “timely countering of widespread myths and disinformation about the health effects of alcohol consumption and alcohol control measures” (p.17), whilst similarly specific issues are not mentioned in the GS.

### Civil society and other entities

The GS highlights the importance of civil society advocates in raising awareness about the impact of harmful alcohol use on individuals, families, and communities, as well as their contribution to mobilising additional commitment and resources for reducing alcohol-related harm (p.19). According to GAAP, these actors are also “invited to scale up their activities in support of global, regional and national awareness and advocacy campaigns”, whilst also countering misinformation provided by the industry (p.17), not mentioned in GS.

The GAAP additionally identifies specific roles for international partners in the United Nations system, intergovernmental organisations, academia and professional associations. All had been identified in GS, with less specificity about their contributions. Finally, the GAAP also encourages collaborations among international organisations, civil society, professional associations, and experts.

## Cross-cutting meta-theme: Harms to health by commercial activities and policy interference by the alcohol industry need to be restricted

In the GS, the alcohol industry is portrayed as a potential partner in alcohol policymaking. It acknowledges the industry as important players in their various roles as developers, producers, distributors, marketers, and sellers. The strategy encourages the industry “to consider effective ways to prevent and reduce harmful alcohol use within their core roles” (p.20). The GS recognises the alcohol industry as one of several participants with distinct roles to play. The GAAP, in contrast, emphasises the need for protection from policy interference by these same actors and urges Member States to ensure that policy measures are protected from the influence of commercial interests. It uses the language of “economic operators in alcohol production and trade”, to whom it addresses proposed measures separately from other interests, also identifying transnational corporations and the alcohol industry, albeit very much less prominently. GAAP now identifies that any activities by commercial actors should be “stringently” within core roles (p.14). GAAP makes it clear that using industry resources to secure policy influence or generate knowledge about alcohol-related harms is inappropriate. Specifically, it asks industry actors to:

Refrain from funding public health and policy-related activities and research to prevent any potential bias in agenda-setting emerging from the conflict of interest and to cease the sponsorship of scientific research on the public health dimensions of alcohol consumption and alcohol policies and its use for marketing or lobbying purposes (p.21).

This is a clear departure from what the GS asked of economic operators, indicating a profound shift in how the global public health community sees the policy roles and ethical responsibilities of the alcohol industry. This is identified as a cross-cutting meta-theme because it is interwoven with all three presented core themes.

As reported in Table 1, the connection between the meta-theme and the sub-themes varied. The sub-themes relating to theme 3 (greater specification of public health policy actors’ roles and responsibilities) exhibited the most overlap with the meta-theme. For example, both policy documents provided some guidance for how such policy actors should engage with commercial interests. According to the GAAP, Member states are expected to ensure that policy development is “protected from the interference of commercial interests” (p.13). Yet concerns about industry influence are relevant even if implicit when considering the GAAP documents’ governing principles (e.g., public health interests should guide the design of policies and interventions) and the persistence of specific governance challenges (e.g., the need to increase decision-makers’ attention to alcohol-related harm).

Within the GS, there is some acknowledgement of the tensions between the alcohol industry’s economic interests and the policy aims of reducing alcohol consumption and harm. Beyond this, the GS tends to emphasise “appropriate engagement” and consensus-building, portraying industry actors as partners and crucial “stakeholders” in reducing alcohol consumption (p.10). In contrast, the GAAP explicitly identifies the “influence of powerful commercial interests in policy-making and implementation” (p.5). The GAAP links limited priority attached to alcohol-related harm to the specific activities of the industry, including “low awareness” of alcohol-related harms and “commercial messaging and poorly regulated marketing of alcoholic beverages” (p.3).

Another key departure between the GS and GAAP relates to the treatment of competing interests. The GS calls for giving “appropriate priority” to public health concerns but also emphasises the need to “take into account other goals, obligations, including international legal obligations, and interests.” The GS presents public health measures as potentially harmful to “economic interests and […] government revenues” (p.7). The GAAP, on the other hand, warns that alcohol policy development at all levels requires “safeguarding… from alcohol industry interference” (p.5). The shifting tone of language and focus indicates a significant change in approach.

The GS regards the effective “balancing [of] different interests” as a governance challenge (p.7). While the GS does not provide explicit guidance on how to strike this balance, the absence implies that these conflicting interests should be reconciled through traditional political processes (e.g., representation of interests, negotiation and consensus if possible). There is a discernible shift in language between the two documents, with terms such as “balancing” and “partnership” with the alcohol industry now entirely absent from the GAAP.

The GAAP treats industry interference as an ongoing and critical governance issue, part of the problem rather than the solution. This perspective is exemplified by one operational principle of the GAAP: “The development of public policies to reduce the harmful use of alcohol should be protected, in accordance with national laws, from commercial and other vested interests that can interfere with and undermine public health objectives” (p.35). The GAAP also requires that Member States “manage conflicts of interests” (p.19).

The GAAP also pays less attention to policy issues that have historically been raised by the alcohol industry. GAAP is less concerned about illicit production and trade; it acknowledges that this is an issue but one that needs to be treated separately. GAAP thus leaves this issue to Member States to develop responses according to the extent of the problem encountered, thus recognising that it is a problem, but also not letting it have disproportionate prominence in global-level strategy discussions.

While the GAAP’s treatment of the alcohol industry is notably different from the GS, it does, nonetheless still identify some key roles for economic operators which are deviant cases, as arguably not strictly within core roles, and thus which exhibit tensions with the main finding of change. According to Action Area 2, for example, the alcohol industry is “invited […] to develop and enforce self-regulatory measures related to marketing and advertising.” This is suggested to be done either independently from Member States (i.e., self-regulation) or in collaboration with the establishment and implementation of statutory regulations (i.e., co-regulatory framework). Co-regulation suggests that industry will continue to participate in the policy process as it implies at the very least communication, if not agreement, on policy. This is particularly problematic since it may perpetuate the legitimation efforts of industry actors to stay involved in shaping alcohol policies on alcohol marketing and more broadly. As such, it risks undermining the policy interference content of GAAP, and time will tell how far such content is seized upon by industry actors.

## Discussion

This study analyses significant recent developments in alcohol policymaking at the global level. By comparing the two key policy documents, after their endorsement by the WHA, this is a study of the apex of alcohol policy globally. Our analysis identifies several core themes that demonstrate key continuities – for instance, in the fundamental principles guiding policy action. Also, both documents acknowledge similar governance challenges that may be intrinsic to alcohol policymaking, albeit with some notable differences in emphasis. We find that the envisaged roles of different policy actors have remained broadly consistent, though now with improved definitions, with the particular exception of the alcohol industry. This study helps address the strikingly under-developed literature on alcohol policy development at the global level. Researchers have amassed a deeper understanding of the barriers and opportunities that high-impact, cost-effective policies confront in the policymaking process at the national level (for example, [32, 33]). Our study, however, offers the first comparative analysis of the two global-level policy documents, examining continuities and change.

The most significant change between the documents is the GAAP’s treatment of the alcohol industry. Policymakers now see safeguarding alcohol policymaking from the influence of commercial interests as a key governance priority. While the GS acknowledges the potential for conflicts of interest, the GAAP provides more explicit guidance that the limited implementation of the GS can be attributed, at least in part, to the alcohol industry’s actions. The study suggests that the political power of the alcohol industry at the global level may be waning in important ways [41]. Our analysis shows clear departures in how the alcohol industry, and specifically the legitimacy of its participation, is now perceived. This is consistent with developments at the national level, with the alcohol industry’s influence on policy having ebbed in some contexts, with governments implementing population-level alcohol policy measures, including alcohol pricing policies [32, 33, 44]. The ways in which the GAAP attributes the lack of progress to policy interference by industry appear to make it unlikely that the future course of alcohol policy development can be changed, without a major rupture. This seems unlikely given the increased ambition reflected in the targets set in the GAAP and their location within the countdown to the United Nations (U.N.) goals for 2030.

Existing evidence of the industry’s influence at the global level is, however, largely indirect. According to one study of the GAAP process, there is sparse evidence to suggest that industry arguments significantly influenced modifications to the action plan [8]. The authors, however, drew attention to the different drafts that existed throughout the process. Particularly relevant to the findings of the present study, there is evidence that there were changes in the text on industry actors throughout this process [8]. In other institutional contexts, such as the World Trade Organization (WTO), it may be unsurprising for national governments to echo the policy concerns articulated by the alcohol industry, given the nature of their remit. Although also a U.N. agency, the WHO is importantly different given the primacy of health [75]. There is thus an important research gap to be filled with historical scholarship on the extent to which the GS was originally substantially influenced by the major alcohol companies. That is certainly what the alcohol industry sought to do [11, 12], though such direct evidence, although somewhat historical in nature, would arguably be compelling today. Evidence of this nature may stimulate further alcohol policy developments at all levels. That may be challenging to obtain given the “behind closed doors” nature of the political compromises forged between different Member States and other stakeholders [76]. Further research is also needed to investigate the GAAP process and how far different institutional settings mediate the impact of the alcohol industry’s framing strategies on, and other interventions in, alcohol policy. The ongoing implementation of the GAAP at the national level across the world provides another high priority target for research.

The impetus in alcohol policy development appears now to be heading in a clear direction of travel. This raises the stakes in the contest between global health and the alcohol industry [14]. Such a situation raises clear questions about how the major alcohol companies will respond. The major companies that lead the alcohol industry have clear strategic choices to make. These actors may continue to follow the path of their counterparts in tobacco, with whom they have been closely connected in long-term strategy development [77] and major company ownership [78]. That means circumventing traditional policymaking structures as well as undermining them, and attempting to influence public attitudes towards alcohol, the alcohol industry, and alcohol policy [79]. It is 25 years since the alcohol industry was warned that if it was not to become a pariah like the tobacco industry, then it should stop acting in the same ways [80]. That message was not heeded at the time and there is no evidence that it has been at any time since then.

The present findings also contribute conceptual nuance to the study of commercial actors in global health institutions. The CDoH literature is beginning to underscore power imbalances between national governments and multinational commercial actors, including the global alcohol producers [60, 81], thus making global institutions, and WHO in particular, more important. Political science approaches have begun to enrich studies of the alcohol industry and alcohol policy [33, 37, 43–45, 54, 82–84], and will be useful in this regard. The CDoH literature directs attention toward political practices and institutional processes [55, 84–86]. Our findings suggest complementary avenues for exploration, beyond those previously discussed. Policy learning frameworks, for instance, theorise how policymakers refine their beliefs through experiential learning, analysis, and social interactions, subsequently translating these insights into policy decisions [87]. Although most such research focuses on technical learning [88], studies of political learning explain how policymakers draw on their previous experiences to devise new political strategies for advancing policy goals [89, 90]. In studies of alcohol policy, political learning offers a promising analytical perspective. Research at the national policymaking level has identified how lesson-drawing was instrumental in helping policymakers withstand opposition from industry to minimum unit pricing and other public health measures [43, 46]. In the present study, there is direct evidence of political learning. Following their experience with the GS, policymakers developed a carefully nuanced view of industry interference as a hurdle to global alcohol policy development, and adjusted the action plan accordingly, securing the support of Member States in so doing. Thus, there appears obvious potential for conceptual cross-fertilisation to further understanding of this area of public health policy and the powerful commercial actors who seek to shape it in their own interests.

The study has several limitations that must be borne in mind. First, the analysis only examines two policy documents, potentially overlooking other relevant policy changes in generating inferences about developments at the global level. For instance, the WHO places significant emphasis on the SAFER initiative [23, 68]. Our analysis does not examine SAFER in any depth. Second, as a thematic analysis of data sources in the public domain, it must undoubtedly be the case that supplementing the present study with research interviews involving actors would provide valuable additional insights into the nature and extent of developments at the global level, including decision-making processes. At the same time, there is value in providing careful analysis and comparison of the products of these policymaking processes. Third, we have used thematic analysis as our approach. It may be the case that other analytic approaches will produce different findings. We suggest the nature of this study lends itself to replication studies, and we also invite the employment of other analytic approaches. Fourth, while our analysis finds the GAAP to constitute a significant policy change, its impact on global health will largely hinge on the political will of Member States to implement what they have agreed, and if not, how further policy failure is reckoned with later on. There may be a risk that we overstate the importance of both the GS and GAAP, but we regard this possibility as so small that it should be discounted. Agreements of this nature can foster policy action by serving as commitment devices for national governments, as well as helping mobilise the public health community to pursue securing adequate resources and monitor the implementation of the formally made commitments [91–94].

Furthermore, there is a clear need for research to examine the extent to which national governments and other actors adhere to their designated roles and responsibilities, particularly regarding the exclusion of industry actors from global and national alcohol public health policymaking. Studies are also needed on how far alcohol industry tactics influence policy agendas, policymakers, and the public outside of the formal processes of policymaking [37, 55, 73, 95, 96].

## Conclusion

The adoption of the GAAP appears to herald an important development in global alcohol policy, in the context of careful comparison with the GS, though it is unclear how fully it may be implemented. The alcohol industry may continue to obstruct the implementation of effective interventions which regulate the alcohol market, and if so, the ambitious targets set for 2030 will not be reached. Perhaps the key advance in GAAP over the longer term lies in clearly identifying that the alcohol industry should not be seen as any kind of partner in public health policymaking and its commercial activities are so harmful to health that we should not expect major progress in global health in this area without effective regulation.

## Data Availability

All data generated or analysed during this study are included in this published article.

## Abbreviations

FCTC: Framework Convention on Tobacco Control (FCTC)
GAAP: Global Alcohol Action Plan
GS: Global Strategy for Reducing the Harmful Use of Alcohol
WHA: World Health Assembly
WHO: World Health Organization
WTO: World Trade Organization
EB: World Health Organization Executive Board
